# Tribological Properties of High-Entropy Alloys under Dry Conditions for a Wide Temperature Range—A Review

**DOI:** 10.3390/ma14195814

**Published:** 2021-10-05

**Authors:** Ashish K. Kasar, Kelsey Scalaro, Pradeep L. Menezes

**Affiliations:** Department of Mechanical Engineering, University of Nevada, Reno, NV 89557, USA; akasar@nevada.unr.edu (A.K.K.); kscalaro@nevada.unr.edu (K.S.)

**Keywords:** high-entropy alloys, wear, friction, hardness, self-lubrication

## Abstract

High-entropy alloys (HEAs) are composed of multiple elements with equimolar or near equimolar composition that have superior mechanical and tribological properties. In this article, we present a review on the tribological performance of HEAs. The tribological properties of different HEAs systems have been evaluated, and it has been found that the wear rate strongly depends on the crystal structure of the phases. The most common structures are face-centered cubic (FCC), body-centered cubic (BCC), and dual-phase (FCC + BCC) alloys due to the high entropy of mixing instead of forming intermetallic phases. In general, HEAs with a BCC structure showed superior hardness and wear properties compared to FCC and FCC + BCC alloys. The lesser wear rate of HEAs with a BCC structure is attributed to the reductions in ductility, resulting in strong but brittle alloys. In addition to the crystal structure, the effect of temperature on the tribological performance of the HEAs is also discussed, which highlights their potential applications for high temperatures. Moreover, various other factors such as grain size, formation of an oxide layer, and wear mechanisms are discussed.

## 1. Introduction

Since the inception of material science, metallurgists have been striving to create stronger and lighter metals with other tailored properties. Alloying has been a boon to achieve properties superior to single elements. For example, the addition of a small amount of carbon to iron led to the varieties of steel and cast iron with superior strength, ductility, corrosion resistance, etc. However, the quantity of the alloying element in the traditional alloys is limited in terms of quantity and alloying elements. This led to the foundational work of Yeh et al. [1] on a new field of materials called high-entropy alloys (HEAs) with expanded alloying quantity and elements. In the past 20 years, the development and characterizations of these materials have garnered significant attention due to their superior properties, including high hardness, high strength, excellent ductility, good wear, and high corrosion resistance [2,3]. These superior properties are achieved through leveraging the characteristics of HEAs that set them apart from traditional alloys. The defining aspect of HEAs is that they are made by tailoring a limitless combination of at least five primary elements used in large proportions. The use of five or more elements in large proportions leads to an increase in configurational entropy of mixing, resulting in the term HEAs. HEAs have specific characteristics including sluggish diffusion, lattice torsion, and the cocktail effect [4].

**Figure 1:**
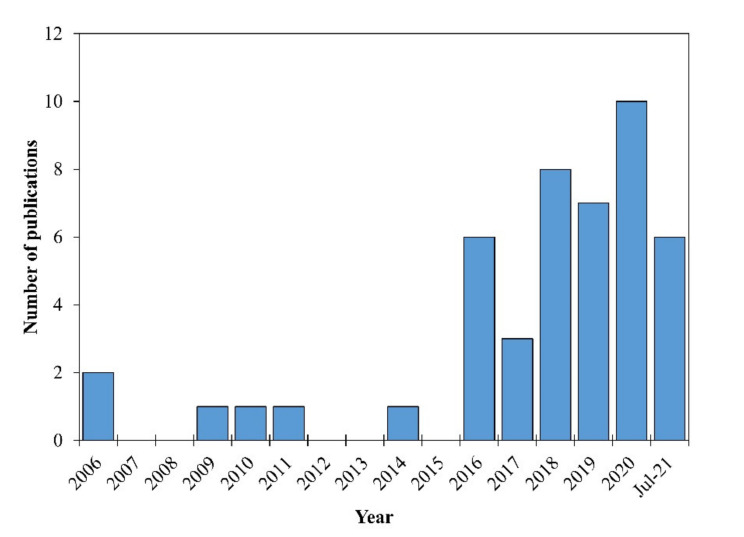
Tribological properties of high-entropy alloy ingots publication distribution.

In the past five years, publications concerning HEAs have dramatically increased as the field of material science seeks to develop application-specific alloys. Additionally, books and literature reviews have been published in hopes of starting to collect and organize the rapidly growing body of literature from a broad spectrum of HEAs [5,6,7,8,9] according to the specific criterion or properties of HEAs, such as nanocrystalline [10], refractory [11] phase transition and corrosion properties [12], fatigue, fracture properties of HEAs [13], high-temperature wear [9,14], and additive manufacturing of HEAs [15]. Along with the influx of general development and characterization work for HEAs, there has also been an increase in tribological studies to understand the friction and wear performance of these alloys. Figure 1 presents a histogram of the published dates of papers used in the tribological comparison portion of this study and goes back as far as 2006, just two years after Yeh’s foundational paper. The papers were found using the search features of the Web of Science. The increase in publications about tribology in the past five to six years aligns with the overall rise in high-entropy alloy papers as described by Vaidya et al. [6]. It is worth noting that the collection of papers for this literature review ceased during August 2021, which explains the relatively low number of papers included for that year. Despite this increase in popularity, there is a noticeable lack of literature reviews to collect and synthesize existing tribological work. The wear of materials is one of the leading causes of mechanical failure and warrants more research to characterize materials so the industry can make educated design choices. This literature aims to provide information that can be used to support metallurgists as they pursue additional research concerning the development of HEAs specifically for tribological applications.

This literature review exclusively focuses on dry sliding tribological properties of ingot cast HEAs. Studies considering tribological properties of a high-entropy alloy in different environments were included, but only the dry sliding comparison case is presented in this review. The high-temperature tribological performance of the HEAs is also discussed. Materials included in this review are categorized based on their crystal structures, face-centered cubic (FCC), body-centered cubic (BCC), and FCC + BCC phases, since these are the most common crystalline phases in ingot cast HEAs.

## 2. Design of HEAs

**Figure 2:**
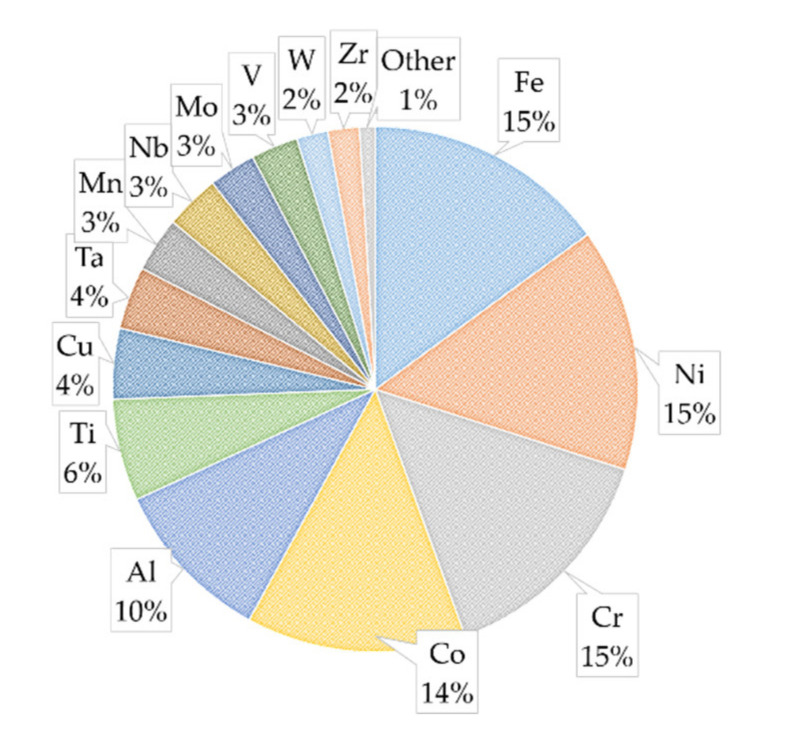
High-entropy alloy base element distribution.

HEAs are defined as the combination of five or more elements in equal or near equal ratios (5–35 atomic %). The other definition of HEAs is based on the configurational entropy of mixing (ΔSmix) that categorized the solid solutions into three categories: high entropy (>1.61R), medium entropy (0.69R–1.61R), and low entropy (<0.69R) alloys [16]. However, there are few HEAs that follow the compositional definition but have lower ΔSmix ~1.37R. By considering these two definitions, the alloys with ΔSmix > 1.5R can be considered as HEAs [17]. By following these definitions, there are around 30 elements currently employed in HEAs’ development, and additional elements may be added in proportions less than 5% similar to traditional alloys [1,18]. Figure 2 illustrates the distribution of base elements from the materials included in this review. To avoid repetitiveness, only base materials were included in this figure. Here, the term “base material” refers to the initial or basic high-entropy alloy being studied or modified; for example, an HEA such as AlCoCrFeNi that has five versions tested with different amounts of carbon added would only be documented as one material in this figure. The most common elements used in the included papers are Fe, Ni, Cr, and Co. These three elements are also included in the highly studied Cantor alloy, CrMnFeCoNi [19]. This alloy and its five base elements are often used as a starting point for developing other HEAs by either replacing an element or adding small portions of an additional alloying element [9]. It is also important to highlight that out of the 40 different base HEAs in this review, 31 of them are either a Cantor alloy or a variation of it. Some studies directly focused on the tribological properties of the Cantor alloy, while a handful further modify the Cantor alloy by adding ratios of Ti, C, Nb, Al, or Cu to it. Another common modification of the Cantor alloy for high-entropy alloy development is replacing one base element with another; replacing Mn with Al was a common variation in the publications included in this review.

**Figure 3:**
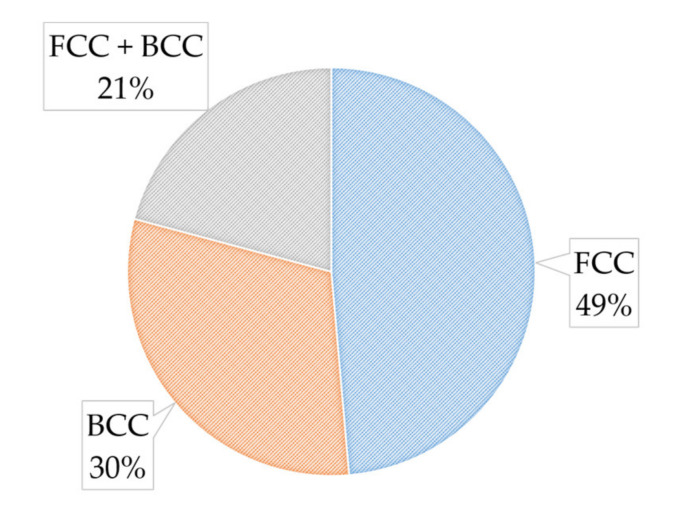
High-entropy alloy major phase distribution.

HEAs typically form simple crystalline phases including FCC, BCC, or a combination of the two. Simple ordered phases such as B2 and L1 phases and complex ordered phases such as laves also form within cast high-entropy alloy ingots, which are less common. This literature categorized the HEAs on BCC, FCC, and FCC + BCC phases. Figure 3 illustrates the distribution of phases for the materials included in this paper. Most papers described how these phases were determined by using X-ray diffraction (XRD). The most common phase type was FCC, which was also the same phase as observed in the Cantor alloy (CrMnFeCoNi) and many of its variations. Some studies included investigating what ratios of a base element or an additional element would cause the major phase of the high-entropy alloy to shift from FCC to BCC [20,21,22]. The reason BCC phases are largely considered more desirable than FCC phases is described in later sections of this review. 

The combination of multiple elements in relatively large amounts lends HEAs to have a few core characteristics, including the high entropy effect, lattice torsion, sluggish diffusivity, and the cocktail effect. The slugging diffusion of atoms is due to the large variety of atoms in the lattice, leading to inconsistent bonding and energies, making it difficult for atoms to move around and fill vacancies [20]. This gridlock promotes nano-sized particles, which increases strength, promotes stability at high temperatures, reduces creep, and lends HEAs to be used as diffusive barrier coatings. The lattice distortion core effect considers how the different sizes of elements cause distortion in the lattice structure, which creates internal strain energies. These impede the movement of dislocations in the lattice, which supports solid solution strengthening and reduces electrical and thermal conductivity. The cocktail effect considers how the combination of elements and the properties result in something that may be non-intuitive or a combination of properties per the rule of mixtures. It is the overall effect of the combination of elements and considers the composite element’s individual properties as well as how they interact with each other. 

The replacement of base elements or the addition of other elements to a high-entropy alloy is performed with specific property goals in mind. This review article presents the common addition or replacement materials for the Cantor alloy and their influences on the phases and properties. The addition of V has been shown to increase the hardness and, conversely, the wear rate. This beneficial effect stops after a certain percentage is added [23,24]. The inclusion of Al and Ti similarly increased hardness and reduced wear rates [20,25]. Adding Bi reduced the coefficient of friction (COF) [26], the addition of Ti and Zr both improved wear rates [27], and Mo increased hardness [28]. The addition of carbon and Cr_3_C_2_ both increased the hardness but consequently reduced the ductility of the part and, at some ratios, was ill-advised due to excessive brittleness [29,30]. The addition of some elements has seemingly no effect on material properties, as is the case of Pb [31]. The balancing of positive and negative effects of elements is necessary for developing and modifying new HEAs for specific conditions, and choices must be purposefully made. A few programs even exist that help predict properties for different combinations before they are cast, which aids in development.

## 3. HEAs: Manufacturing Methods

A major advantage of HEAs is that they can be produced using the existing technologies and this does not require any individualized processing techniques or equipment. The current manufacturing techniques widely used for research are discussed in this section. This is not to say that the manufacturing of HEAs is an inexpensive endeavor. They are still relatively costly to make compared to other alloys, but the fact that no new processes or machines need to be developed is a big selling point. Manufacturing HEAs presents a couple of challenges since combining so many different elements in such high proportions means the different densities, atom sizes, and melting temperatures make it difficult to produce consistent and homogenous results. 

The most common methods include arc melting, induction melting, and spark plasma sintering. Other methods mentioned include hot press sintering and air melting. The most common method in the publications included in this review used arc melting or vacuum arc melting to produce their test ingots. Spark plasma sintering was the second most popular method. Induction melting made up a relatively small portion of research used for tribological properties but is still rather common for manufacturing HEAs and should be discussed for that reason. Almost all included papers only used one manufacturing method, but a few compared methods. These papers compared the tribological properties of one material produced via vacuum arc melting or spark plasma sintering [32], vacuum induction melting or additive manufacturing [33], and vacuum arc melting or air melting [34]. The specific methods used to manufacture an HEA ingot play a significant role in forming the specific crystalline structures and precipitates forming in the material matrix. In the literature, little attention was paid to this aspect that may influence tribological properties, leaving ample room for future research.

### 3.1. Arc Melting

**Figure 4:**
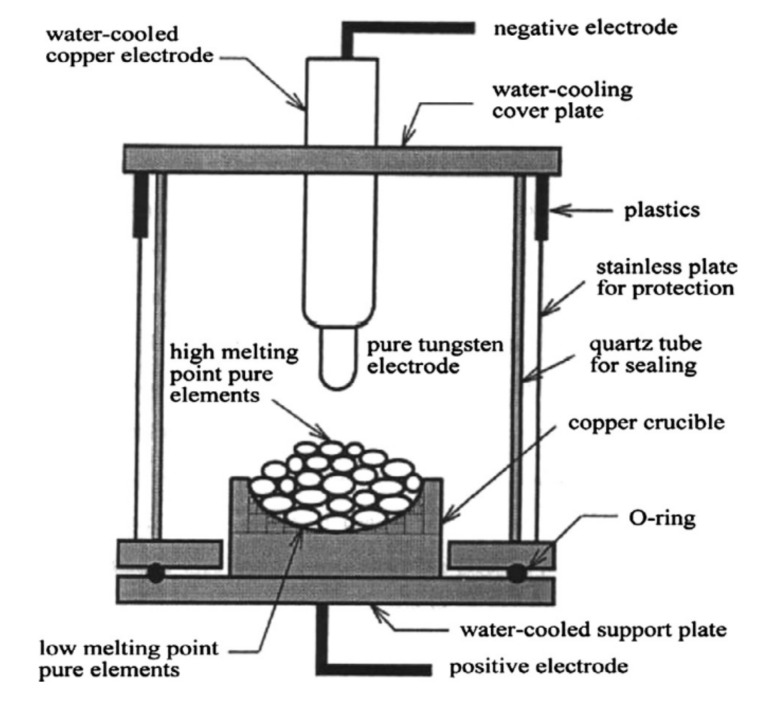
Vacuum arc melting system [36].

Arc melting and vacuum arc melting (VAR) are popular melting and casting processes for producing high-entropy alloy ingots [35] and was the most commonly used method for the studies included in this review. This method is desirable due to its ability to produce relatively homogeneous ingots that can be manufactured in small lab-sized quantities and much larger industrial sizes. The detailed research to industry methodology promotes the fabrication of HEAs using this method [5]. During arc melting, pure elements in the desired proportions are measured and added to a water-cooled crucible often made of copper or ceramic (see Figure 4). For vacuum arc melting processing, the chamber is evacuated and filled with an inert gas such as argon to reduce oxidation during the melting and cooling of the ingots. An arc is struck between a tungsten electrode and the crucible contents to melt the material, which promotes turbulent mixing, often aided by an electromagnet stirrer. The ingots are then remelted multiple times to promote homogeneity and reduce segregation. The prepared alloy is then allowed to solidify and cool within the crucible, forming a “button” ingot, or is poured into a water-cooled mold [5]. The high melting temperatures typical of this method can process high-melting point materials such as titanium and remove trace elements. A study by Moravcikova-Gouvea et al. [32] found that high-entropy alloy ingots made using the arc melting method are better than the spark plasma sintering for applications that require high wear resistance, but intrinsic brittleness is not an issue.

### 3.2. Induction Remelting

**Figure 5:**
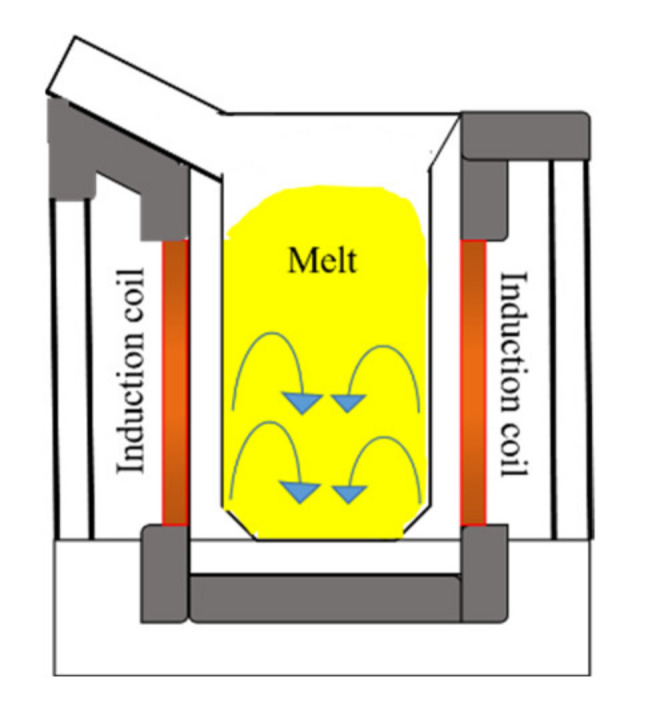
Induction melting system.

Similar to arc melting, induction melting is popular since it can be scaled from research to industrial applications. This method was not frequently used for the studies described in this paper but is a popular method for developing and manufacturing HEAs. The lower operating temperatures make this an ideal method for processing materials that would otherwise evaporate out during arc melting [36]. Material is added to a graphite crucible as solid pieces of raw material or as powder compacts of pre-mixed material. An induction coil is wrapped around the entire crucible to melt the material through electromagnetic induction (refer to Figure 5). As the material heats and melts via Joule heating, eddy currents are generated, which vigorously stir the melt and produce good mixing and homogeneity [37]. After being remelted multiple times, the fabricated alloy is poured into water-cooled mold and allowed to cool. Since HEAs are prone to oxidation, vacuum induction remelting (VIR) is often used where the entire process happens within an inert gas environment. The melting and cooling rates for this method are difficult to control but are considered easier than arc melting, making it a desirable choice for controlling microstructure.

### 3.3. Spark Plasma Sintering

Spark plasma sintering (SPS) is a manufacturing method gaining popularity for high-entropy alloy test specimens and represented the second-highest portion of methods used in the papers included in this review. Milled powders are mixed and pressed into dense pellets inside the graphite die before pressure is applied to the material [38]. An AC or DC current is passed through the die and the conductive pellet material, which melts due to Joule heating. Non-conductive materials melt due to the almost instantaneous heating of the mold and surrounding material [39]. This process can be prone to oxidation, but this can be mitigated by milling the pure material powders in an inert atmosphere and conducting the whole sintering process in an argon-filled vacuum chamber. A highly desirable aspect of the spark plasma sintering method is how quickly materials melt and solidify, which limits grain growth and lends itself to the development of well-controlled, nanocrystalline ingots [7]. Due to the applied pressure, the temperatures stay relatively low, and the entire process is fairly cost-effective. The SPS method compared to vacuum arc melting has been found better suited for the development of strong, fine-grained HEAs with a more homogenous microstructure.

## 4. Mechanical and Tribological Properties of the HEAs

The mechanical and tribological properties of the HEAs in this literature review are evaluated by hardness, wear rate, and COF. Mostly, the hardness values were obtained using Vickers hardness test. A variety of wear tests were utilized in the presented papers to measure wear. The most commonly used setups are ball-on-disk, pin-on-disk, and ball-on-block. In each case, a pin or ball counter body is repeatedly moved across a disk or block made of the test materials. A disk rotates relative to a stationary ball or pin for disk tests, creating a circular wear track. A ball is moved relative to the block of test material in a repeated linear motion, creating a wear track for block tests. In each of these methods, the force applied by the pin or block, the speed of movement, and the total distance or time ran are controlled and have an influence on the outcome. One study considered the effects of different speeds [40], others experimented with different loads [27,32,41], and some ran the test for various distances [42,43,44]. The most common counter body materials used for the tribological testing were ceramic materials, including silicon nitride (Si_3_N_4_) and alumina (Al_2_O_3_), followed by various steels, including SKH51, 100Cr6, and GCR15. Most publications only used one counter body material, but a few compared ceramic and steel materials [42,43,44,45,46]. When testing the Cantor alloy against both materials, wear rates were lower for ceramic counter bodies [45,46]. For another set of HEAs that included Mo and Ta along with variations, wear rates were better against steel [42,44,47]. The mechanism is discussed in the following section. The counter body material can have a dramatic effect on wear rates and COF.

Ultimately, all of these tests produce a wear track in the test material. This wear track was then studied to determine the volume loss so it could be used with Archard’s wear equation [48] to solve for the wear rate (see Equation (1)). This equation solves for the wear rate (κ) using the total volume loss of the wear track (ΔV) divided by the sliding distance (s) and the normal load (F). This volume loss was frequently determined by using a 3D optical profilometer which was used to measure the wear track depth and width. Some publications further considered the wear track mechanisms as well as the tribo-layer and oxide layer structure and composition with the use of transmission electron microscopes, scanning electron microscopes, and X-ray photoelectron spectroscopy. The frictional force is also measured during the entire wear test by using a load cell integrated into the tribometer.

(1) $$\kappa =\frac{\Delta V}{s\times F}$$

In Table 1, Table 2, and Table 3, the testing method and relevant parameters are included alongside each material. Authors should discuss the results and how they can be interpreted from the perspective of previous studies and of the working hypotheses. The findings and their implications should be discussed in the broadest context possible. Future research directions may also be highlighted.

### 4.1. Tribological Properties: Room Temperature

A significant characteristic of high-entropy materials is their tendency to form a few simple phases. When HEAs first entered the field of material science, many thought the phases would be plentiful and various intermetallic, but the high configurational entropy prevents this [1,20]. The most common phase in high-entropy materials is BCC, FCC, and a combination of these two. There is a frequent misconception that these phases are the only ones that form in HEAs, but rather, they are the most common. (Refer to Figure 3 to see the general distribution of major phases across the materials included in this review.) This review article focuses on the HEAs with these three phases to understand the tribological mechanism. All materials and test data are included in Table 1, Table 2, and Table 3; each table presents the data collected for one phase. In all three tables, the subscript number represents the molar composition of the HEAs in the first column. The HEAs without subscript numbers suggest equimolar composition. The tribological performance of the HEAs are discussed in the following section.

#### 4.1.1. Tribological Properties of BCC HEAs

Of the phases common in HEAs, BCC is among the most desirable phase [23]. A method for predicting which phases form in a high-entropy alloy is the valence electron concentration (VEC) of the material. For materials with a VEC smaller than 6.87, the dominant phase of the high-entropy alloy is BCC [65]. A desirable quality of BCC phases is the relatively limited slip systems that aid in wear properties and promote brittleness in a material.

**Figure 6:**
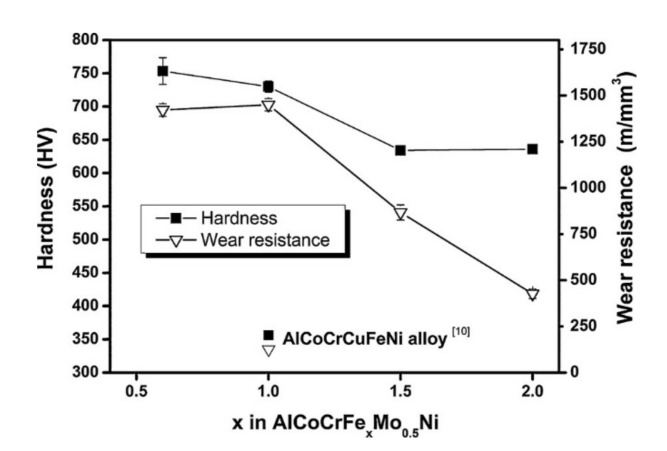
Hardness and wear resistance variation over iron content in the AlCoCrFeMoNi HEA [50].

When developing HEAs, working towards alloys with a BCC major phase is generally seen as desirable. The Cantor alloy, or base alloy, that many HEAs are based on is an FCC material. A significant amount of work considers what ratios of what elements added to this alloy can promote BCC phases. It has been documented that the addition of V [24], Al [20,22,26], Fe [50], and Ti [66] all promote the development and stabilization of BCC phases in HEAs. The addition of these elements to the base high-entropy alloy may increase the likelihood of developing a material with BCC phases, but this comes at the cost of reducing the plasticity of the material. These different property changes must be balanced when developing new materials for specific applications. All data used to construct the figure with BCC phase information are collected in Table 1. For example, the properties AlCoCrFeMo_0.5_Ni HEA alloy with BCC phase can be altered with varying amounts Fe [50]. The increase in iron from 0.6 to 2.0 moles changed the microstructure from dendritic to smaller grains. The presence of Mo also caused the formation of a hard σ phase that resulted in a higher HV hardness of 756. The increase in iron content caused a decrease in hardness due to lesser amount of σ phase. The variation in hardness is shown in Figure 6. The tribological testing parameters are listed in the third entry of Table 1. The wear resistance (inversely proportional to wear rate) decreased with the decrease in hardness which follows the general trend, i.e., wear rate increases with decrease in hardness (Figure 6). The observed trend was due to the higher oxidation tendency of the higher iron content HEA (1.5 and 2 Fe), resulting in a protective oxide layer that lowered the overall wear rate.

Similarly, the effect of Al content in the CoCrFeNiTi0.5Al HEA was studied (entry 4, Table 1) [26]. This study demonstrated that the addition of 0.5 and 1.0 mole of Al increased the hardness by ~9% and 40%, respectively, due to the transition from FCC to harder BCC phases. In this study, no significant wear was observed at room temperature. Table 1 summarizes the other tribological studies on the HEAs with BCC phases. The high-temperature performance of BCC phase HEAs is also listed in Table 1.

#### 4.1.2. Tribological Properties of FCC and FCC + BCC Phase HEAs

**Figure 7:**
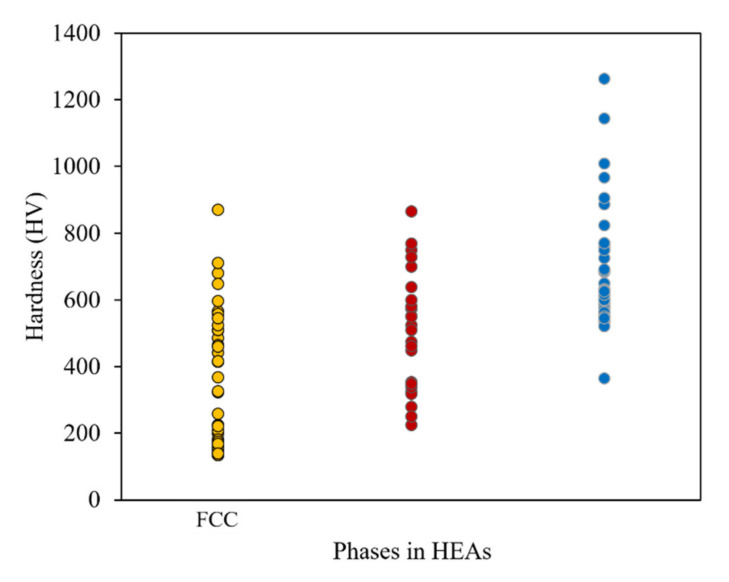
Hardness variation over the major phases of HEAs.

**Figure 8:**
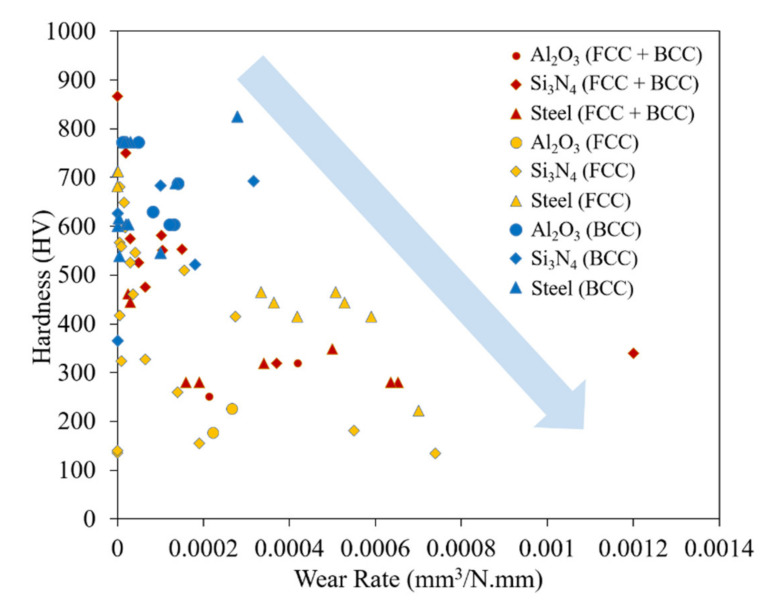
Hardness versus wear rate for HEAs delineated by phase group.

A common phase for HEAs is FCC. The Cantor alloy, CrMnFeCoNi, is dominated by FCC phases [22]. Many of the studies included in this review focus on what elements need to be added or adjusted to promote a shift from FCC to BCC phases. According to Guo’s valence electron consideration theory, a material with a VEC larger than 8 is dominated by simple FCC phases [65]. As a result of atoms within FCC crystals having an increased number of nearest neighbor atoms, there are more slip systems that reduce hardness and consequently wear properties. Despite this, the increase in slip systems does promote an increase in ductility for FCC phase-dominant HEAs. The general hardness of this group of alloys is the lowest of the three-phase groups. Figure 7 illustrates this comparison with the general trend of the material being softer than the other phase groups. The soft material also directly influences wear rate as more material is removed during the tests, leaving much larger wear tracks. Figure 8 illustrates different phase groups’ harness versus wear rates tested against three common counter materials: Al_2_O_3_, Si_3_N_4_, and steel. Figure 8 shows a general trend between the hardness and wear rate with a clear inverse relationship. Additionally, the range of both hardness and wear rate values is larger than for the BCC phase group. The additions of Cu [45,47], and Ni [45] have been shown to promote and stabilize FCC phases. For many applications, better wear properties may be desired, but the FCC phase may have a role when high ductility is required. All data used to construct the figures with FCC phase information are collected in Table 2.

Not all HEAs are solely constructed of only FCC or BCC phases. At times, this is an accident, and may be the result of failed attempts to produce BCC phase materials [21,29,30,64,67]; other times, they may be purposefully developed in hopes of achieving “the best of both worlds” and having some of the high wear properties of BCC phases and the improved ductility of the FCC phases. Per Guo’s valence electron concentration theory, materials with a VEC between 6.87 and 8 tend to have a combination of FCC and BCC phases [65,68]. All data used to construct the figures with FCC + BCC phase HEA information are collected in Table 3. By looking at Table 1 and Table 2, it can be seen that the FCC phase HEAs have higher COF compared to BCC phase HEAs, with a few exceptions.

**Figure 9:**
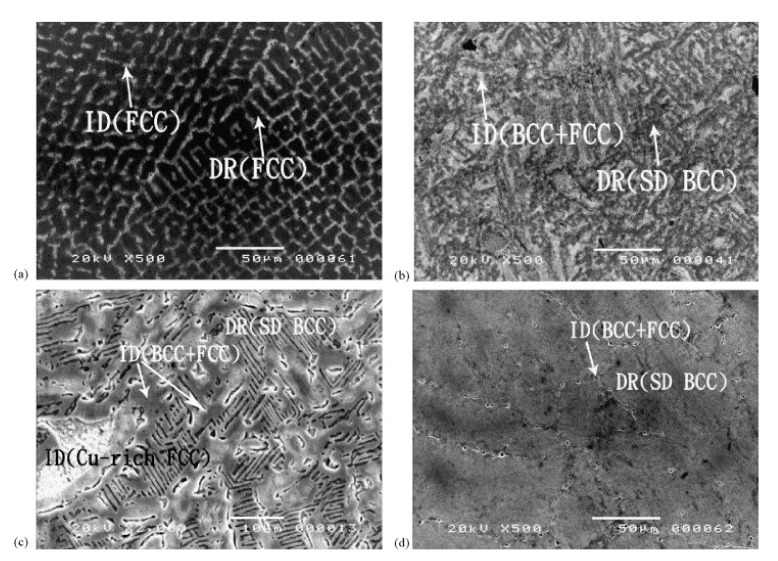
SEM micrographs of AlxCoCrCuFeNi HEAs with x: (**a**) 0.5, (**b**) 1.0, (**c**) 1.0, and (**d**) 2.0 [51].

**Figure 10:**
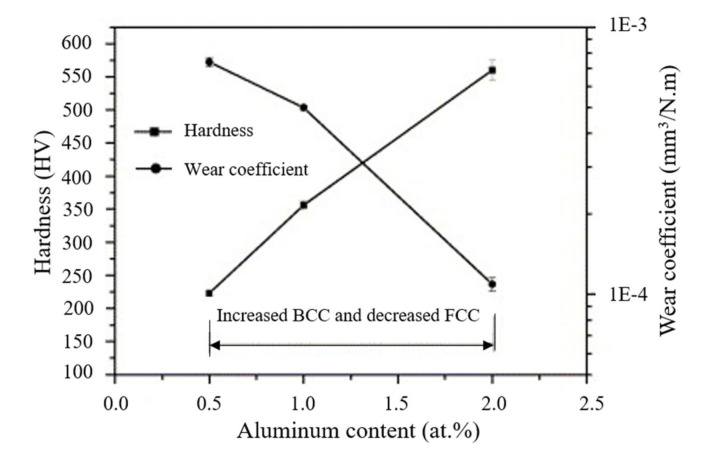
Hardness and wear rate variation over Al content in the AlCoCrCuFeNi HEAs [51].

For example, the increase in the BCC phase in AlxCoCrCuFeNi HEAs was achieved by increasing the Al content from 0 to 2.0 molar ratio [51]. The HEAs prepared by arc melting showed that the interdendritic structure (ID) went from FCC to BCC + FCC with an increase in Al content, whereas the dendritic structures (DR) completely changed from FCC to BCC, as shown in micrographs in Figure 9. The increase in Al content also led to spinodal decomposition of the BCC phase (SD BCC), as highlighted in Figure 9b–d. The change in microstructure and BCC phase evolution also yielded higher hardness and wear coefficient, as shown in Figure 10. The tribological testing details are listed in Table 3, entry 3. The improvement in wear resistance was not only due to an increase in hardness but also due to oxidation at the interface that assisted the wear resistance. A similar phenomenon was also discussed earlier for AlCoCrFeMoNi FCC HEA.

#### 4.1.3. Tribological Properties of HEAs at Elevated Temperatures

**Figure 11:**
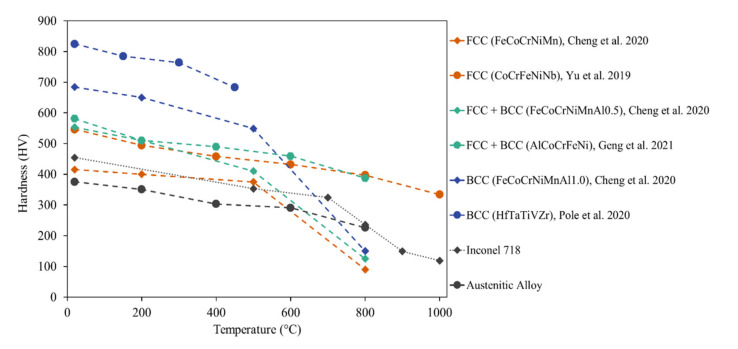
Hardness variation of HEAs over temperature.

HEAs are widely considered good material choices for high-temperature applications. In response to the existing room for the application of these materials, many publications also characterized tribological properties at elevated temperatures. The hardness values of HEAs decrease with an increase in temperature, as shown in Figure 11. However, the BCC phases continue to show relatively higher hardness values compared to that of FCC and FCC + BCC HEAs. Figure 11 illustrates the softening of these materials as temperatures increase; the BCC phases noticeably have the highest hardness values, but all materials soften at close to the same rate. A few alloying materials had been shown to improve the hot hardness, including the addition of Mo [47] and Cu [69]. Hardness values for the HEAs in this review tested beyond room temperature are compared to two common materials (Inconel 718 and Austenitic steel) for perspective. Austenitic steel is a general high-use material, while Inconel 718 is frequently used for high-temperature applications and is noticeably softer at all temperatures than many HEAs.

Despite the reduction in hardness, wear rates for most HEAs showed noticeable improvement. Most materials experienced slight increases in the wear rate, but these decreased after reaching a certain temperature that promoted the formation of oxides. The consideration of oxide formation is a significant aspect of choosing which elements are used in a high-entropy alloy that is intended for high-temperature applications. The addition of W and V [47], Fe [50], C [29], and Nb [60] promoted improvements in wear resistance at high temperatures due to the creation of a strong oxide layer. At certain ratios, the influence of these elements tapered off or no longer aided. When temperatures reached extreme levels and tests were run for sufficient times or distance, oxidative wear mechanisms took over, and the oxidation no longer aided wear resistance. Overall, HEAs seem to be a good choice for elevated temperatures. All temperature data for the HEAs are included in the tables for their specific phase groups.

**Figure 12:**
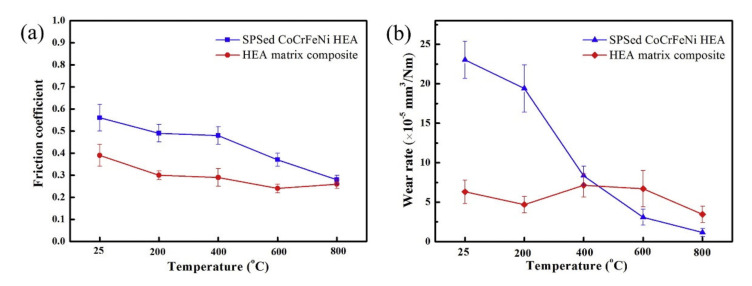
(**a**) COF and (**b**) wear rate for the CoCrFeNi HEA and its composite with MoS_2_ and graphite [70].

From a tribological perspective, in addition to the inherent nature of the HEAs, HEA self-lubricating composites, i.e., the addition of solid lubricants to the HEA matrix, have also been studied. Zhang et al. [70] synthesized the CoCrFeNi HEA alloy with MoS_2_ and graphite as solid lubricants. The HEA composite was prepared by SPS, where Ni-coated graphite and Ni-coated MoS_2_ powders were used. The graphite and MoS_2_ contents were 5 and 8 wt.%, respectively. The tribological tests were conducted against Si_3_N_4_ ball from room temperature to high temperature at 5 N with a sliding velocity of 0.28 m/s. The resulted COF and wear rate were compared with SPSed CoCrFeNi HEA, as shown in Figure 12. With the addition of solid lubricants, the COF was significantly lesser. At the same time, the wear rate above 200 °C was lesser for SPSed HEA compared to HEA self-lubricating composite. The observed behavior was due to the inherent anti-wear properties of the Ni- and Co-based composites, especially at higher temperatures.

Similarly, the other suitable solid lubricants for high-temperature applications, Ag and BaF_2_-CaF_2_, were used in the same HEA matrix of CoCrFeNi [71]. The composites were synthesized by spark plasma sintering after mixing the CoCrFeNi, Ag, and BaF_2_-CaF_2_ powders. The other studies with different solid lubricants such as Ag/h-BN [72] and graphene [73] in the HEA matrix have shown lower COF along with anti-wear properties.

**Figure 13:**
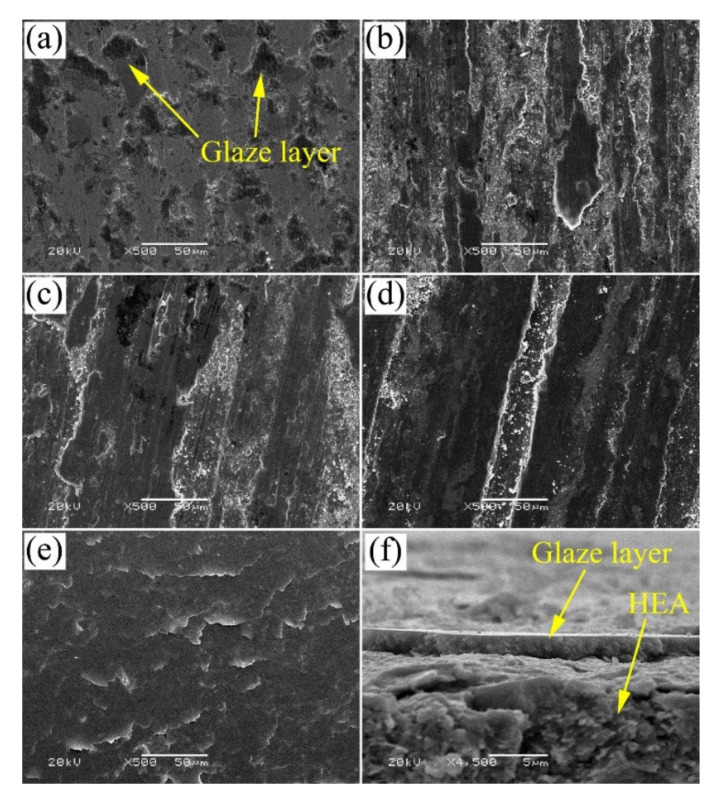
Worn surface of the CoCrFeNiS HEA after tribological tests against Si3N4 at (**a**) 25 °C, (**b**) 200 °C, (**c**) 400 °C, (**d**) 600 °C, and (**e**) 800 °C; (**f**) cross-section of wear track tested at 800 °C [35].

In another study, the role of Cu in the CoCrFeNiCu was evaluated in terms of wear rate at room temperature and 600 °C [27]. The HEAs were synthesized by Arc melting with different Cu concentrations. It was observed that the Vickers hardness increased from 136 to 169 when the Cu content increased from 0 to 1 at.% in the given HEA matrix. The wear rate at 600 °C reduced by 40–45% with the addition of 0.2 to 1 at.% Cu in the CoCrFeNi HEA matrix. The observed behavior was due to the lubricating nature of Cu at higher temperatures that can be easily sheared. The Zhang et al. [35] group also explored the importance of sulfur in the CoCrFeNi HEA matrix where HEA was prepared by spark plasma sintering of the elemental powders in the atomic ratio of 1:1.4:1:1:0.5 for Co:Cr:Fe:Ni:S. The authors confirmed the preferential reaction between Cr and S to form Cr_x_S_y_ by XRD spectrum and SEM micrograph. The tribological test conducted at 5 N against Si_3_N_4_ at different temperatures showed a reduction in COF and wear rate compared to CoCrFeNi HEA with no sulfur. The improved tribological properties were observed due to the formation of Cr_x_S_y_ at the sliding interface shown in the wear track in Figure 13. All the wear tracks were observed with island-shaped glaze layers of the Cr_x_S_y_ phase that suggest smearing of this phase resulted in lower friction and protected the surface from wear.

## 5. Future Scope of the HEAs for Tribological Applications

From the above sections, it can be evaluated that the HEAs have beneficial tribological properties, mainly their high wear resistance. Various studies have demonstrated that the phases in HEAs can be easily tailored by choosing/replacing one of the metal constituents. In addition to the inherent wear resistance properties, their superior higher oxidation resistance and hot hardness, i.e., hardness at the higher temperature, supports tribological applications for adverse temperature conditions. It has also been discussed that the formation of a thin oxide layer reduced the subsequent oxidation and prevented mechanical wear during sliding. Therefore, HEAs are potential candidates to replace existing materials such as Ni-alloys [74,75,76] for high-temperature tribological applications in high-temperature nuclear reactors. However, the oxidation mechanisms for the HEAs are not as simple as conventional metals and alloys due to the presence of multiple elements. As the formation and growth of the different oxide layers depend on the diffusivities and relativities of the individual elements, it brings an opportunity to understand the oxidation behavior of different HEAs, particularly tribo-oxidation. Understanding the tribo-oxidation behavior of HEAs in different environmental conditions will help to design HEAs to boost their applications in moving assemblies.

Moreover, only a few studies have been carried out on the addition of solid lubricants and strengthening phases in the HEAs matrix. There is still a broad scope to study and create a guideline to choose suitable solid lubricants in HEAs to control COF as the solid lubricants have multiple categories [77]. Future studies can be focused on the distribution of solid lubricants in HEAs without reacting with the initial metal constituents.

The other important factor is manufacturing techniques for the HEAs. This review article discusses three manufacturing techniques to fabricate bulk HEAs: arc melting, induction melting, and powder metallurgy. Each process has its advantages and disadvantages, e.g., powder metallurgy process needs pre-alloyed HEAs to fabricate a component, and arc melting requires multiple runs to obtain homogenous composition. However, the processing techniques need to be chosen wisely, mainly to distribute any solid lubricant or strengthening particles from the tribological perspective. So far, the powder metallurgy technique is primarily used to prepare HEAs with dispersed solid lubricants. Hence, it creates an opportunity to explore other techniques to secure an edge over the effective fabrication. One way of fabrication can be additive manufacturing techniques, such as laser powder bed fusion. The utilization of additive manufacturing techniques for HEAs is at an early age, especially HEA composites with solid lubricants. Thus, the utilization of various additive manufacturing techniques for HEAs + solid lubricants can lead to a new category of self-lubricating composites. The effective fabrication also includes cost factors; the HEAs involve costly elements such as Ta, Zr, Co, Nb, Mo, etc. The detailed cost estimation of the HEAs with respect to different metal content are discussed by Fu et al. [78]. The authors suggest that the alloys with Mo, Nb, Bi, and Sn (USD 1–5/mol) would still be expensive than the Ni-based alloys. However, detailed tribological studies need to be performed to estimate the cost and performance matrix of HEAs against the conventional alloys. HEAs also possess a high strength-to-weight ratio compared to other conventional alloys [79]. However, similar to cost estimation, detailed studies are required to estimate the effect of % weight gain while replacing the conventional materials with HEAs since HEAs may include heavy metal constituents. One way to reduce the cost and weight is using an HEA coating instead of using bulk HEAs. It has also been seen that the coating technique, such as magnetron sputtering, resulted in the combination of amorphous and crystalline HEAs [80]. This suggests that the coating can not only reduce the cost but can also provide control over crystallinity.

## 6. Conclusions

The development of HEAs has been an ongoing process since its introduction to the field in 2004. Considering that wear and friction are among the leading causes of part failure, it seems natural that the recent popularity of high-entropy alloy research extends to an increase in studies to understand their tribological properties. This literature review collected publications on the tribological properties of high-entropy alloy ingots undergoing dry-sliding tests at room and elevated temperatures. This review set the foundation for further discussion by first introducing the theory behind HEAs, the manufacturing methods typically used for the production of high-entropy ingots, and common testing methods that were used to determine the tribological properties presented. Careful attention was paid to categorize the content of the publications included, and figures were used to illustrate when papers were published and what material types were studied. Room-temperature tribological properties for HEAs were discussed correlating with the major phase groups typical to these materials: BCC, FCC, and FCC + BCC. BCC phase materials showed superior hardness and wear properties due to their limited slip systems but were also affected by brittleness. FCC phase materials showed inferior wear properties compared to the BCC group but still represented a promising group of materials for specific applications, especially those that require some ductility. FCC + BCC groups had properties that fell between BCC and FCC groups and may serve as an interesting group to further explore in hopes of developing materials that achieve the “best of both worlds”. This review also dedicated space to the exploration of HEAs at elevated temperatures. These materials softened as temperatures increased, but unlike room temperature tests that show softness correlating with increased wear, wear rates were actually lower. This reduction in wear rate is attributed to the oxidation of certain metals at the sliding interface both at room temperature and high temperatures. Furthermore, the effect of an individual element in the one HEA was discussed for multiple HEAs that bring controllability over tribological and mechanical properties, suggesting that the understanding of an individual element is required to design HEAs for specific applications. In addition to the inherent lubricity and anti-wear properties of the HEAs, the self-lubricating HEAs with the addition of solid lubricants have also shown the potential applications of HEAs for lower friction and wear. The discussion and gathered database in this review article can provide a guideline to develop HEAs for tribological applications.

## Figures and Tables

**Table 1:** BCC phase group test procedure information and tribological properties.

Material	Manufacturing Method	Testing Method	Counter Material	Ball/Pin Diameter	Load (N)	Distance or Time	Speed or Frequency	Temperature (°C)	Hardness (HV)	Wear Rate (mm^3^/Nm)	Friction	Reference
AlCoCrFeNi	VAR	Ball-on-Block	Si_3_N_4_	5 mm	3	1200 s	0.2 m/s	Room Temp.	522	0.00018000	0.42	[49]
AlCoCrFeNi	VAR	Pin-on-Disk	Al_2_O_3_	6 mm	15 N	1000 m	100 mm/s	Room Temp.	630	0.00008240	0.73	[22]
								300		0.00005319	0.56	
								600		0.00000924	0.55	
								800		0.00000184	0.42	
								900		0.00000103	0.33	
AlCoCrFe_0.6_Mo_0.5_Ni	Arc smelting	Pin-on-Disk	SKH51	8 mm	29.4	24 h	0.5 m/s	Room Temp.	751	-	0.74	[50]
AlCoCrFe_1_Mo_0.5_Ni									726	-	0.74	
AlCoCrFe_1.5_Mo_0.5_Ni									631	-	0.74	
AlCoCrFe_2_Mo_0.5_Ni									632	-	0.74	
Al_2_CoCrCuFeNi	VAR	Pin-on-Disk	SKH51	8 mm	29.4	64,800 m	0.5 m/s	Room Temp.	546	0.00010000	0.32	[51]
AlCoCrFeNiTi_0.5_	SPS	Reciprocating Wear	Al_2_O_3_	10 mm	26	900 s	40 Hz	Room Temp.	750		1.22	[52]
								500			1.12	
								650			1.13	
								800			1.06	
								900			0.85	
AlCrFeNiTi	VAR	Pin-on-Disk	GCrl5 Steel	9 mm	20	1000 m	0.8 m/s	Room Temp.	616	0.00000260	0.55	[53]
AlCrFeNiTiMn_0.5_									539	0.00000510	0.55	
CoCrFeNiTi_0.5_Al_0.5_	Induction Melting	Ball-on-Disk	WC	6 mm	10	216 m		Room Temp.	968		0.35	[26]
								250			0.45	
								500			0.55	
CoCrFeNiTi_0.5_Al								Room Temp.	1264			
								250			0.37	
								500			0.47	
Co_0.5_CrCu_0.5_FeNi_1.5_AlTi_0.4_	Arc Melting	Pin-on-Disk	Si_3_N_4_	3.175 mm	0.25	200 m	8.5 mm/s	Room Temp.	626	0.00000003	0.55	[54]
								100			0.6	
								300			0.25	
TiNbCrCoAl								Room Temp.	365	0.00000011	0.6	
								100			0.9	
								300			0.6	
CuMoTaWV	SPS	Ball-on-Disk	Alloy Steel	9.33 mm	5	200 m	0.1 m/s	Room Temp.	600	0.00000050	0.5	[47]
			Si_3_N_4_					Room Temp.	600	0.00400000	0.45	
								200		0.00230000	0.6	
								400		0.00500000	0.67	
								600		0.04500000	0.54	
FeCoCrNiMnAl_1_	Hot Press Sintering	Ball-on-Disk	Si_3_N_4_	5 mm	10	15 min	450 RPM	Room Temp.	684	0.00010000	0.31	[20]
								200	650	0.00012500	0.4	
								500	580	0.00012000	0.275	
								800	150	0.00250000	0.19	
HfTaTiVZr	VAR	Ball-on-Disk	Si_3_N_4_	3 mm	50	190 m	5 Hz	Room Temp.	693	0.00031600	0.25	[55]
								150	635	0.00072100	0.28	
								300	642	0.00024800	0.27	
								450	622	0.00023800	0.25	
TaTiVWZr								Room Temp.	825	0.00027900	0.32	
								150	785	0.00079000	0.35	
								300	764	0.00011100	0.32	
								450	683	0.00011100	0.3	
MoTaNbZrTi	VAR	Ball-on-Disk	100Cr6 Steel	6 mm	5	400 m	10 cm/s	Room Temp.	688	0.00013500	0.5	[44]
						1000 m				0.19000000	0.5	
			Al_2_O_3_			400 m				0.00014100	0.75	
						1000 m				1.35000000	0.75	
MoTaNbVTi	VAR	Ball-on-Disk	100Cr6 Steel	6 mm	5	400 m	10 cm/s	Room Temp.	604	0.00002542		[42]
						1000 m				0.00001957		
						2000 m				0.00002520		
			Al_2_O_3_			400 m				0.00013200		
						1000 m				0.00012960		
						2000 m				0.00012100		
MoTaWNbV	VAR	Ball-on-Disk	Al_2_O_3_	6 mm	5	1000 m	10 cm/s	Room Temp.	722	1.57000000		[56]
			100Cr6 Steel							2.32000000		
MoTaWNbV	VAR	Ball-on-Disk	100Cr6 Steel	6 mm	5	400 m	10 cm/s	Room Temp.	772	0.00002980	0.75	[43]
						1000 m				0.00002320	0.8	
						2000 m				0.00001670	0.8	
			Al_2_O_3_			400 m				0.00004840	0.65	
						1000 m				0.00001570	0.8	
						2000 m				0.00001050	0.8	
TiZrNbHf	VAR	Pin-on-Disk	GCr15 Steel Ball	6 mm	5	5400 mm	3 Hz	RT	225	0.002407	0.61	[57]
Al_0.25_TiZrNbHf									280	0.002074	0.58	
Al_0.5_TiZrNbHf									325	0.002055	0.55	
Al_0.75_TiZrNbHf									375	0.001740	0.60	
Al_1_TiZrNbHf									419	0.001629	0.58	

**Table 2:** FCC phase group test procedures and tribological properties.

Material	Manufacturing Method	Testing Method	Counter Material	Ball/Pin Diameter	Load	Distance or Time	Speed or Frequency	Temperature (°C)	Hardness (HV)	Wear Rate (mm^3^/Nm)	Friction	Reference
Al_1_Co_2_Cr_3_Fe_3_Ni_1_ (FCC + σ + A2/B2)	SPS	Ball-on-Disk	Si_3_N_4_	6.43 mm	5	340 m	0.19 m/s	Room Temp.	509	0.00015522	0.75	[58]
								200	458	0.00020199	0.67	
								400	428	0.00037326	0.65	
								600	397	0.00013511	0.70	
								800	336	0.00003800	0.57	
Al_0.2_Co_1.5_CrFeNi_1.5_Ti	SPS	Ball-on-Plate	AISI 52100	9.51 mm	1.2	30 min	2 Hz	Room Temp.	712	0.00000008	0.74	[32]
					5					0.00000110	0.60	
	VAR				1.2				682	0.00000002	0.67	
					5					0.00000003	0.66	
Al_0.3_CoCrFeNi	Arc Melting	Pin-on-Disk	Si_3_N_4_	3.175 mm	0.25 N	200 m	8.5 mm/s	Room Temp.	135	0.00000009	0.80	[54]
								100			0.70	
								300			0.60	
Al_0.3_CuCrFeNi_2_								Room Temp.	140	0.00000007	0.80	
								100			0.80	
								300			0.60	
Al_0.5_CoCrCuFeNi	VAR	Pin-on-Disk	SKH51	8 mm	29.4 N	64,800 m	0.5 m/s	Room Temp.	222	0.00070000	0.50	[51]
Al_0.25_CoCrFeNi	Arc Melting	Ball-on-Disk	Si_3_N_4_	5.5 mm	10 N	30 min	0.084 m/s	Room Temp.		0.00014000	0.72	[59]
								100		0.00023000	0.65	
								200		0.00027700	0.61	
								300		0.00034000	0.57	
								400		0.00036000	0.57	
								500		0.00034000	0.52	
								600		0.00035000	0.51	
CoCrFeMnNi (FCC + σ phase)	Arc Melting	Pin-on-Disk	Al_2_O_3_	6mm	5 N	1000 m	100 mm/s	Room Temp.		0.00044148		[41]
								600		0.00001580		
								800		0.00004732		
					15 N			Room Temp.		0.00028983		
								600		0.00001768		
								800		0.00003429		
CoCrFeNiMn	SPS	Ball-on-Disk	Si_3_N_4_	6 mm	5 N	30 min	0.28 m/s	Room Temp.	1.81	0.00055000	0.62	[30]
								200		0.00035000	0.59	
								400		0.00007541	0.44	
								600		0.00006494	0.42	
								800		0.00000463	0.37	
CoCrFeNiMn–Cr_3_C_2_ (10 wt.%)								Room Temp.	323	0.00000878	0.46	
								200		0.00004283	0.63	
								400		0.00002282	0.50	
								600		0.00000578	0.48	
								800		0.00000546	0.35	
CoCrFeNiMn- Cr_3_C_2_ (20 wt.%)								Room Temp.	417	0.00000454	0.44	
								200		0.00001053	0.56	
								400		0.00003708	0.53	
								600		0.00003228	0.62	
								800		0.00003704	0.35	
CoCrFeNiMn–Cr_3_C_2_ (40 wt.%)								Room Temp.	681	0.00000447	0.44	
								200		0.00000748	0.33	
								400		0.00004329	0.66	
								600		0.00001014	0.57	
								800		0.00000743	0.40	
CoCrFeNi	SPS	Ball-on-Disk	GCr15 Steel	6.35 mm	5 N	3.6 m	6 mm/s	Room Temp.	415	0.00059000	0.76	[28]
					50 N					0.00041800	0.63	
CoCrFeNiMo_0.1_					5 N				443	0.00052800	0.75	
					50 N					0.00036400	0.61	
CoCrFeNiMo_0.3_					5 N				465	0.00050700	0.71	
					50 N					0.00033400	0.59	
CoCrFeNiTi_0.5_ (FCC + σ)	Induction Melting	Ball-on-Disk	WC	6 mm	10 N	216 m		Room Temp.				[26]
								250				
								500				
CoCrFeNiNb_0.5_ (FCC + Laves phase)	Arc Melting	Pin-on-Disk	Si_3_N_4_	6.55 mm	5 N	30 min	0.188 m/s	Room Temp.	546	0.00004095	0.82	[60]
								200	494	0.00005959	0.58	
								400	458	0.00007319	0.38	
								600	432	0.00003007	0.54	
								800	397	0.00001971	0.37	
								1000	334			
CoCrFeNiNb_0.65_ (FCC + Laves phase)								Room Temp.	597	0.00001833	0.77	
								200	562	0.00004988	0.49	
								400	526	0.00007504	0.38	
								600	501	0.00004984	0.52	
								800	436	0.00000069	0.60	
								1000	391			
CoCrFeNiNb_0.8_ (FCC + Laves phase)								Room Temp.	649	0.00001603	0.70	
								200	604	0.00004759	0.45	
								400	578	0.00008533	0.46	
								600	552	0.00005825	0.60	
								800	497	0.00000064	0.70	
								1000	429			
CoCrFeMnNiC	Mechanical alloying + SPS	Ball-on-Disk	Si_3_N_4_	5 mm	20 N	100 m	4 Hz	Room Temp.	327	0.00006500	0.76	[61]
CoCrFeMnNiC_0.3_									460	0.00003600	0.78	
CoCrFeMnNiC_0.6_									566	0.00000470	0.67	
CoCrFeMnNiC_0.9_									558	0.00000860	0.69	
CoCrFeMnNiC_1.2_									525	0.00003000	0.77	
CoCrFeNiCu	Arc Melting	Pin-on-Disk	Not Specified	8 mm	100 N			Room Temp.	136	0.00002293		[27]
								600		0.00002333		
CoCrFeNiCu_0.2_								Room Temp.	143	0.00001893		
								600		0.00002013		
CoCrFeNiCu_0.4_								Room Temp.	153	0.00001893		
								600		0.00001700		
CoCrFeNiCu_0.6_								Room Temp.	158	0.00002520		
								600		0.00001467		
CoCrFeNiCu_0.8_								Room Temp.	160	0.00001353		
								600		0.00001413		
CoCrFeNiCu_1_								Room Temp.	169	0.00001507		
								600		0.00001320		
Co_1.5_CrFeNi_1.5_Ti_0.5_	VAR	Ball-on-Disk	Al_2_O_3_	6 mm	5 N	1000 m	0.1 m/s	Room Temp.	368	0.00113000		[45]
			100Cr6 Steel							0.00052000		
CoCrFeMnNi	VAR	Pin-on-Disk	Al_2_O_3_	6 mm	15 N	1000 m	100 mm/s	Room Temp.	255	0.00026630	0.61	[22]
								300		0.00007836	0.57	
								600		0.00001573	0.55	
								800		0.00001837	0.43	
								900		0.00002193	0.35	
Al_0.3_CoCrFeNi								Room Temp.	176	0.00022324	0.64	
								300		0.00008199	0.55	
								600		0.00001760	0.51	
								800		0.00001702	0.44	
								900		0.00001556	0.34	
FeCoCrNiMn	Hot Press Sintering	Ball-on-Disk	Si_3_N_4_	5 mm	10 N	15 min	450 RPM	Room Temp.	415	0.00027500	0.25	[20]
								200	400	0.00037500	0.25	
								500	375	0.00030700	0.25	
								800	90	0.00005000	0.25	
AlCoCrFeNi_2.1_	VAR	Reciprocating	Si_3_N_4_	6.35	30	6300 mm	1 Hz	500	-	100	0.36	[62]
								600	-	650	0.47	
								700	-	1100	0.64	
								800	-	1800	0.37	
								900	-	1600	0.40	

**Table 3:** FCC + BCC phase group test procedures and tribological properties.

Material	Manufacturing Method	Testing Method	Counter Material	Ball/Pin Diameter	Load	Distance or Time	Speed or Frequency	Temperature	Hardness (HV)	Wear Rate (mm^3^/Nm)	Friction	Reference
Al_0.3_CrFe_1.5_MnNi_0.5_	VAR	Pin-on-Disk	SKH51		29.4 N	64800 m	0.5 m/s	Room Temp.	462	0.00002510	0.56	[34]
	Air melting								444	0.00002930	0.51	
Al_1.3_CoCuFeNi_2_	VAR	Ball-on-Block	Si_3_N_4_	5 mm	3 N	1200 s	0.2 m/s	Room Temp.	340	0.00120000	0.63	[61]
Al_1_CoCrCuFeNi	VAR	Pin-on-Disk	SKH-51 Steel	8 mm	29.4 N	5400 m	0.5 m/s	Room Temp.	349	0.00050000	0.48	[51]
Al_0.6_CoCrFeNi	VAR	Pin-on-Disk	Al_2_O_3_	6 mm	15 N	1000 m	100 mm/s	Room Temp.	251	0.00021328	0.57	[22]
								300		0.00007927	0.56	
								600		0.00001627	0.51	
								800		0.00002369	0.39	
								900		0.00003022	0.32	
AlCrCuFeNi_2_	VAR	Ball-on-Block	Si_3_N_4_	5 mm	5 N	30 min	0.2 m/s	Room Temp.		0.00216300	0.35	[63]
					10 N					0.00047800	0.45	
					15 N					0.00030500	0.25	
AlCoCrFeNi_2.1_	VAR	Ball-on-Disk	Al_2_O_3_	6 mm	4.9 N	30 min	0.28 m/s	room temp	319	0.00042000	0.59	[46]
			Si_3_N_4_							0.00037000	0.54	
			GCr15 Steel							0.00034000	0.57	
			SiC							0.00009700	0.5	
								200	306	0.00021200	0.68	
								400	282	0.00041400	0.69	
								600	270	0.00061800	0.73	
								800	258	0.00081900	0.34	
								900	197	0.00092200	0.29	
AlCoCrFeNi (FCC + A_2_/B_2_ + σ)	SPS	Ball-on-Disk	Si_3_N_4_	6.43 mm	5 N	340 m	0.19 m/s	Room Temp.	551	0.00010540	0.79	[58]
								200	489	0.00016656	0.7	
								400	469	0.00031334	0.63	
								600	428	0.00010688	0.66	
								800	377	0.00001911	0.43	
Al_1.3_Co_1.15_Cr_1_Fe_1_Ni_1.3_ (FCC + A_2_/B_2_ + σ)								Room Temp.	581	0.00010160	0.69	
								200	510	0.00013830	0.63	
								400	489	0.00018005	0.615	
								600	459	0.00004840	0.632	
								800	387	0.00001675	0.384	
Al_0.6_CoCrFeNi	VAR	Ball-on-Block	GCr15 Steel	5 mm	5 N	1800 s	2 Hz	Room Temp.	280	0.00065200	0.542	[40]
							3 Hz			0.00063600	0.536	
							4 Hz			0.00015900	0.365	
							5 Hz			0.00019000	0.402	
CoCrFeNiAl_0.25_Ti_0.75_	Arc Melting	Pin-on-Disk	Si_3_N_4_	3.175 mm	0.25 N	200 m	8.5 mm/s	Room Temp.	866	0.00000003	0.6	[54]
								100			0.8	
								300			0.55	
FeCoCrNiMnAl_0.5_	Hot Press Sintering	Ball-on-Disk	Si_3_N_4_	5 mm	10 N	15 min	450 RPM	Room Temp.	553	0.00015000	0.32	[20]
								200	510	0.00020000	0.32	
								500	410	0.00017500	0.275	
								800	125	0.00003500	0.19	
FeCoNiCuAl	SPS	Ball-on-Disk	Si_3_N_4_	6 mm	10 N	30 min	0.5 m/s	Room Temp.	475	0.00006500	0.53	[64]
								600		0.00076000	0.55	
FeCoNiCuAl–TiC(10wt.%)								Room Temp.	525	0.00004890	0.67	
								600		0.00005440	0.46	
FeCoNiCuAl–TiC(20wt.%)								Room Temp.	575	0.00002920	0.67	
								600		0.00001730	0.39	
FeCoNiCuAl–TiC(30 wt.%)								Room Temp.	750	0.00001979	0.6	
								600		0.00001680	0.35	

## Data Availability

Data sharing is not applicable to this article.
